# Dietary Protein Intake in Young Children in Selected Low-Income Countries Is Generally Adequate in Relation to Estimated Requirements for Healthy Children, Except When Complementary Food Intake Is Low^1^,^2^,^3^

**DOI:** 10.3945/jn.116.239657

**Published:** 2017-02-15

**Authors:** Joanne E Arsenault, Kenneth H Brown

**Affiliations:** 4 Program in International and Community Nutrition, University of California, Davis, CA; and; 5 Bill & Melinda Gates Foundation, Seattle, WA

**Keywords:** protein, amino acids, PDCAAS, dietary adequacy, protein quality

## Abstract

**Background:** Previous research indicates that young children in low-income countries (LICs) generally consume greater amounts of protein than published estimates of protein requirements, but this research did not account for protein quality based on the mix of amino acids and the digestibility of ingested protein.

**Objective:** Our objective was to estimate the prevalence of inadequate protein and amino acid intake by young children in LICs, accounting for protein quality.

**Methods:** Seven data sets with information on dietary intake for children (6–35 mo of age) from 6 LICs (Peru, Guatemala, Ecuador, Bangladesh, Uganda, and Zambia) were reanalyzed to estimate protein and amino acid intake and assess adequacy. The protein digestibility–corrected amino acid score of each child’s diet was calculated and multiplied by the original (crude) protein intake to obtain an estimate of available protein intake. Distributions of usual intake were obtained to estimate the prevalence of inadequate protein and amino acid intake for each cohort according to Estimated Average Requirements.

**Results:** The prevalence of inadequate protein intake was highest in breastfeeding children aged 6–8 mo: 24% of Bangladeshi and 16% of Peruvian children. With the exception of Bangladesh, the prevalence of inadequate available protein intake decreased by age 9–12 mo and was very low in all sites (0–2%) after 12 mo of age. Inadequate protein intake in children <12 mo of age was due primarily to low energy intake from complementary foods, not inadequate protein density.

**Conclusions:** Overall, most children consumed protein amounts greater than requirements, except for the younger breastfeeding children, who were consuming low amounts of complementary foods. These findings reinforce previous evidence that dietary protein is not generally limiting for children in LICs compared with estimated requirements for healthy children, even after accounting for protein quality. However, unmeasured effects of infection and intestinal dysfunction on the children’s protein requirements could modify this conclusion.

See corresponding commentary on page 725.

## Introduction

Previous research indicates that infants and young children in low-income countries (LICs)^6^ generally consume greater amounts of protein from complementary foods than published estimates of protein requirements, assuming average amounts of breast milk consumption (1–3). Therefore, it is widely believed that growth restriction, which is common in these settings, is not due to protein deficiency. However, previous assessments did not account for protein quality, which incorporates information on the specific mix of amino acids in complementary foods and breast milk and the digestibility of ingested protein, and did not use currently accepted methods for assessing total nutrient intake adequacy of populations, based on usual intake distributions after adjusting for within-individual variation (4). In addition, a recent FAO report on the evaluation of dietary protein adequacy suggested that protein quality should be evaluated with the use of the specific amino acid content of ingested protein and digestibility factors based on ileal digestibility, which are lower than the fecal digestibility factors used in the past (5). Thus, previous assessments may have overestimated protein adequacy of the diets.

The concept of protein quality evaluation has been discussed mainly with respect to individual foods, but there are few instances of its application to whole diets of children in LICs. The protein digestibility–corrected amino acid score (PDCAAS) was introduced in a 1989 FAO/WHO Expert Consultation to evaluate foods or diets based on the amino acid content in relation to a reference protein and the digestibility of the dietary protein (6). The method was updated and endorsed in a subsequent WHO/FAO/United Nations University Expert Consultation on protein requirements (7). The PDCAAS can be applied to whole diets by calculating the sum of amino acids from all foods while correcting for digestibility, comparing with a reference scoring pattern, and adjusting the crude total dietary protein intake to estimate the amount of protein in the diet that is available for metabolism and tissue synthesis. The protein in breast milk and animal-source foods has a high PDCAAS because it is highly digestible and is composed of more-than-adequate amounts of all of the essential amino acids, whereas most plant-based foods tend to have a lower PDCAAS because the protein is less digestible and contains lower amounts of some essential amino acids, particularly lysine (in cereals) and sulfur-containing amino acids (in legumes). Therefore, diets in LICs that are highly dependent on staple cereals and low in animal-source foods generally have a lower PDCAAS, but the amount of available protein also depends on the amount of crude protein consumed.

To assess the protein adequacy of children’s diets in LICs, taking into account protein quality, we have reanalyzed data from previous studies of young children’s dietary intake amounts to calculate the PDCAAS and the available protein intake with the use of fecal digestibility factors and the amino acid content of foods. Because of the currently limited data on ileal digestibility factors for individual amino acids for a broad range of foods, we simulated the impact of reductions of fecal digestibly factors by 5% and 10% on dietary protein adequacy.

## Methods

### 

#### Description of data.

Individual-level dietary data were obtained from the investigators or data curators of 7 studies conducted in low-income communities of 6 countries in 3 regions: Latin America (Peru, Guatemala, and Ecuador); South Asia [Bangladesh (2 studies, hereafter referred to as Bangladesh-1 and Bangladesh-2)]; and Sub-Saharan Africa (Uganda and Zambia). Details of the studies have been reported previously (8–14). A brief synopsis of the data sets and diet methodologies are provided in **Table 1**. The ages of the children included in the present analyses ranged from 6 to 35 mo. Dietary intake values were assessed for the following age groupings based on age-specific protein requirements: 6–8 mo, 9–11 mo, 12–17 mo (or 12–23 mo), and 24–35 mo. Four of the studies (Peru, Guatemala, Bangladesh-1 and Bangladesh-2) assessed dietary intake by direct observation and food weighing, including breast milk intake. Three studies (Ecuador, Uganda, and Zambia) assessed dietary intake of foods by 24-h recall, so only children who were not breastfeeding were included in the present analyses, with total intake of breastfed children not measured. Five of the studies contained multiple days of dietary studies on at least a subset of individuals. Some of the studies were longitudinal (Peru, Guatemala, and Ecuador), and for these studies children may have been included in analyses for >1 age category.

**TABLE 1:** Characteristics of analysis data sets

Reference	Region	Country and community description	Years of survey	Children, *n*	Age range, mo	Includes breastfeeding children	Diet method	Description of diet studies and number of children^1^
8	Latin America	Peru, periurban area (Trujillo)	2003–2004	302	6–15	Yes	Daytime weighed observation, including breast milk	Longitudinal, 1–2 d every 3 mo, 1–3 times during a 6-mo period; age 6–8 mo: 1 d (134), 2 d (154), and 4 d (3); age 9–11 mo: 1 d (117), 2 d (117), and 4 d (8); and age 12–17 mo: 1 d (2) and 2 d (95)
9	Latin America	Guatemala, periurban (Guatemala City)	1997–1998	260	6–15	Yes	Daytime weighed observation, including breast milk	Longitudinal, 2 d every 3 mo, 1–3 times during a 6-mo period; age 6–8 mo: 1 d (55), 2 d (125), 3 d (24), 4 d (18), and 6 d (1); age 9–11 mo: 1 d (37), 2 d (99), 3 d (23), 4 d (20), 5 d (8), 6 d (1), and 7 d (1); and age 12–17 mo: 1 d (9), 2 d (40), 3 d (37), 4 d (75), 5 d (6), 6 d (8), and 7 d (2)
10	Latin America	Ecuador, 2 rural areas (coastal and highlands) and 1 periurban area (Quito)	2003–2005	500	12–35	No	24-h recall	Longitudinal, 1 d every ∼1 mo, 1–5 times during a 6-mo period; age 12–23 mo: 1 d (73) and 2 d (175); age 24–35 mo: 1 d (69) and 2 d (247)
11	South Asia	Bangladesh-1, 1 rural district	1999	123	6–11	Yes	Daytime weighed observation, including breast milk	Cross-sectional, 1 d
12	South Asia	Bangladesh-2, 2 rural districts	2007–2008	222	24–35	Yes	Daytime weighed observation, including breast milk	Cross-sectional, 1–2 d; 1 d (26) and 2 d (196)
13	Africa	Uganda, 3 rural districts	2007–2009	168	12–35	No	24-h recall	Cross-sectional, 1–2 d; age 12–23 mo: 1 d (47) and 2 d (19); 24–35 mo: 1 d (121) and 2 d (58)
14	Africa	Zambia, 2 rural districts	2009	107	24–35	No	24-h recall	Cross-sectional, 1 d

1 In the longitudinal studies, children may be represented in several age categories; the number of children with the specified number of days of diet studies in each category are in parentheses.

**TABLE 2:** Child anthropometric characteristics by age group^1^

	Children, *n*	Age, mo	Weight, kg	Length, cm	Weight-for-age *z* score	Weight-for-length *z* score	Length-for-age *z* score	Stunted, %
Aged 6–8 mo								
Peru, all	291	7.5 ± 0.8	7.6 ± 0.8	66.0 ± 1.9	−0.61 ± 0.85	0.44 ± 0.96	−1.56 ± 0.59	19.6
Guatemala, all	223	6.9 ± 0.9	7.0 ± 0.9	64.2 ± 2.5	−1.12 ± 1.00	−0.06 ± 0.93	−1.66 ± 0.95	34.5
Guatemala, BF	180	6.8 ± 0.9	6.9 ± 0.9	64.1 ± 2.4	−1.12 ± 1.04	−0.09 ± 0.92	−1.62 ± 0.97	33.9
Guatemala, non-BF	43	7.0 ± 0.9	7.0 ± 0.8	64.2 ± 2.7	−1.13 ± 0.83	0.04 ± 0.97	−1.80 ± 0.86	37.2
Bangladesh-1, BF	53	7.7 ± 0.8	6.7 ± 0.8	66.0 ± 2.7	−1.75 ± 1.05	−1.16 ± 1.16	−1.45 ± 1.08	32.7
Aged 9–11 mo								
Peru, all	242	10.2 ± 0.8	8.3 ± 0.9	69.6 ± 2.0	−0.62 ± 0.82	0.24 ± 0.91	−1.55 ± 0.63	21.0
Guatemala, all	189	9.8 ± 0.8	7.6 ± 0.9	67.3 ± 2.5	−1.25 ± 1.03	−0.16 ± 0.94	−2.00 ± 0.98	48.9
Guatemala, BF	141	9.8 ± 0.7	7.6 ± 1.0	67.3 ± 2.6	−1.31 ± 1.07	−0.22 ± 0.94	−2.02 ± 1.01	49.7
Guatemala, non-BF	48	9.9 ± 0.8	7.8 ± 0.9	67.4 ± 2.3	−1.06 ± 0.91	0.02 ± 0.96	−1.94 ± 0.87	46.8
Bangladesh-1, BF	70	10.3 ± 0.9	7.3 ± 0.9	68.7 ± 2.9	−1.71 ± 1.02	−1.08 ± 0.96	−1.71 ± 1.11	35.3
Aged 12–17 mo								
Peru, all	97	13.2 ± 0.8	8.9 ± 0.9	72.1 ± 1.9	−0.63 ± 0.77	0.26 ± 0.89	−1.77 ± 0.59	28.9
Guatemala, all	177	13.2 ± 0.5	8.3 ± 1.0	70.9 ± 2.7	−1.25 ± 1.06	−0.26 ± 0.95	−2.12 ± 0.99	53.7
Guatemala, BF	117	13.2 ± 0.6	8.2 ± 1.1	70.7 ± 2.9	−1.42 ± 1.07	−0.41 ± 1.07	−2.22 ± 1.05	59.0
Guatemala, non-BF	60	13.2 ± 0.6	8.6 ± 1.0	71.3 ± 2.3	−0.91 ± 0.96	0.04 ± 0.99	−1.92 ± 0.85	43.3
Aged 12–23 mo								
Ecuador, non-BF	248	19.9 ± 2.8	9.6 ± 1.0	77.0 ± 3.0	−1.18 ± 0.74	−0.17 ± 0.83	−2.16 ± 0.65	54.8
Uganda, non-BF	47	19.4 ± 3.5	10.2 ± 1.8	78.3 ± 4.6	−0.61 ± 1.45	0.04 ± 1.10	−1.46 ± 1.47	10.8
Aged 24–35 mo								
Ecuador, non-BF	316	28.7 ± 2.9	11.1 ± 1.0	83.4 ± 2.8	−1.21 ± 0.72	−0.16 ± 0.77	−2.00 ± 0.65	44.3
Bangladesh-2, all	222	29.4 ± 3.3	10.6 ± 1.5	83.9 ± 4.9	−1.78 ± 1.03	−0.90 ± 0.88	−2.05 ± 1.29	53.2
Bangladesh-2, BF	140	28.7 ± 3.2	10.3 ± 1.3	83.3 ± 4.5	−1.88 ± 0.96	−1.03 ± 0.84	−2.09 ± 1.18	53.6
Bangladesh-2, non-BF	82	30.5 ± 3.3	10.9 ± 1.6	84.8 ± 5.5	−1.60 ± 1.12	−0.69 ± 0.91	−1.99 ± 1.46	52.4
Uganda, non-BF	121	29.7 ± 3.3	11.8 ± 1.7	85.6 ± 4.6	−0.80 ± 1.10	0.16 ± 1.00	−1.67 ± 1.21	28.1
Zambia, non-BF	107	31.3 ± 5.5	11.9 ± 1.6	84.4 ± 5.1	−0.87 ± 1.01	0.45 ± 0.92	−2.11 ± 1.37	59.8

1 Values are means ± SDs. BF, breastfed.

Anthropometric data for the children were available at the time of the diet studies in 5 studies (Peru, Bangladesh-1 and -2, Uganda, and Zambia). For the other 2 studies, anthropometric data from the nearest time before and after the diet study was used to estimate the weight and height at the time of the diet study based on the interpolated daily rate of weight change during the interval between available anthropometric measurements. Anthropometric *z* scores were calculated with the use of WHO growth reference data (15).

#### Assessment of dietary protein and amino acid intake.

Amino acid values were assigned to all foods with the use of data from the USDA Standard Reference database, version 27 (16), and the International Minilist (17). If the protein value of the food item in the original publication, based on the food composition table used by that study, differed from the protein value in the amino acid source data, the amino acid values were adjusted according to the protein value of the original report, based on milligrams of amino acid per gram of protein. Breast milk amino acid values were obtained from the WHO/FAO/United Nations University (7). Fecal digestibility factors were assigned to each food item, primarily from the FAO (6), and supplemented with information from the literature (18–21).

Protein intake values were corrected for protein quality and digestibility with the use of the PDCAAS method to estimate the amount of protein available for use by the body (7). First, digested protein and amino acid intake from each food was calculated as the product of the amount consumed and the digestibility factor of that food. The crude protein and the digestible protein and amino acid intake values were summed for each child-day. For each child-day, a weighted digestibility factor was calculated as the proportion of digested protein to crude protein. For each amino acid, the sum of digested amino acid intake values per gram of digested protein intake was divided by the amino acid requirement per gram of protein, with the use of the reference pattern for ages 6 mo to 3 y as suggested by the 2013 FAO report on protein quality assessment (5). The lowest ratio (i.e., the proportion of amino acid intake to requirement) was identified for each child-day, and this is the amino acid score. The PDCAAS truncated at 1 was calculated as the product of the amino acid score and the weighted digestibility factor. Finally, the amount of available protein intake for each child was calculated by multiplying his or her original (crude) protein intake by the PDCAAS.

The adequacy of protein and amino acid intake was assessed based on requirements on a per-kilogram body weight basis (5). Each child’s available protein and digested amino acid values were divided by his or her body weight at the time of the diet study. The percentage of children with an intake below the average requirement was calculated with the use of National Cancer Institute (NCI) macros (22) to estimate usual intake distributions at each time point while adjusting for within-person variation in intake. For the 2 studies that contained only 1 d of intake per child, PC-SIDE software (via the Intake Monitoring, Assessment and Planning Program module) (23) was used because it allows for the use of external estimates of variance to estimate a usual intake distribution. The variance estimates from Peru were used to account for day-to-day variability of intake in Bangladesh-1 because of the age and breastfeeding prevalence similarities, and estimates from Uganda were used for Zambia because of age and dietary similarities. Usual intake distributions (median, 25th percentile, and 75th percentile) for energy, protein, and amino acids were obtained from the NCI macros or PC-SIDE.

Child characteristics of each cohort were described by means and SDs. The percentages of protein intake from breast milk, animal-source complementary foods, and plant-source foods were calculated by dividing the sum of the protein intake values from each source for all person-days by the sum of the total protein intake values for all person-days within each cohort. It was not possible to determine accurately which children had usual protein intake values below the Estimated Average Requirement (EAR), because many days of intake are necessary to estimate an individual’s usual intake, and the statistical modules (NCI and Intake Monitoring, Assessment, and Planning Program) only give an estimate of the population distribution of usual intake. Nevertheless, for descriptive purposes, we examined differences in dietary factors between children who had protein intake below the EAR or greater than or equal to the EAR by using generalized linear regression or nonparametric analyses (Wilcoxon sign-rank test). Statistical significance between these 2 groups was set at 0.05.

Ileal digestibility factors are currently available for just a few foods, so we simulated the potential impact of ileal digestibility on protein adequacy by repeating the analyses with 5% and 10% lower digestibility than the fecal digestibility factors, except for breast milk, which remained at 100% digestibility. All analyses were conducted with the use of SAS version 9.3.

## Results

A summary of anthropometric characteristics for each of the study and age cohorts is provided in **Table 2**. Some of the original studies included a specific cutoff for length-for-age *z* score (LAZ) as a criterion for selection into the study; specifically, children in the Peru study had an initial LAZ less than −0.5, and children in the Ecuador study had an LAZ less than −1.25 compared with reference data available at the time of these studies. The prevalence of stunting was >50% in the Guatemalan (aged 12–17 mo), Ecuadoran (aged 12–23 mo), Bangladeshi-2 (aged 24–35 mo), and Zambian (aged 24–35 mo) children.

The mean usual energy intake of the children ranged from a low of 76 kcal/kg body weight for Peruvian infants (aged 6–8 mo) and Bangladesh-1 infants (aged 9–11 mo) to a high of 117 kcal/kg body weight for Zambian children (**Table 3**). Mean energy requirements for infants (aged 6–12 mo) ranged from 78 to 81 kcal/kg body weight (24), which indicates that a few of the infants in Peru and Bangladesh-1, as well as breastfeeding Guatemalan children, may have had inadequate energy intake.

**TABLE 3:** Usual dietary intake distributions of energy and protein in children aged 6–35 mo from 6 low-income countries^1^

	Children, *n*	Energy, kcal/d	Energy, kcal · ^−1^ · ^−1^	Protein, g/d	Available protein, g/d	Available protein, g · kg^−1^ · d^−1^	EAR,^2^g · kg^−1^ · d^−1^	Less than EAR, %
Aged 6–8 mo								
Peru, all	291	563 (487, 649)	75 (64, 86)	12.8 (9.8, 16.7)	12.8 (9.7, 16.6)	1.69 (1.28, 2.20)	1.12	15.7
Guatemala, all	223	602 (527, 685)	87 (76, 100)	12.9 (10.0, 16.6)	12.8 (9.8, 16.5)	1.85 (1.40, 2.41)	1.12	11.3
Guatemala, BF	180	576 (515, 639)	84 (74, 94)	11.1 (9.3, 13.2)	11.0 (9.2, 13.0)	1.59 (1.30, 1.93)	1.12	12.0
Guatemala, non-BF	43	738 (644, 835)	106 (91, 121)	23.3 (18.1, 28.4)	23.0 (17.7, 28.1)	3.30 (2.53, 4.06)	1.12	1.3
Bangladesh-1, BF	53	532 (460, 606)	76 (68, 89)	9.1 (7.7, 10.6)	8.9 (7.6, 10.2)	1.27 (1.12, 1.49)	1.12	24.4
Aged 9–11 mo								
Peru, all	242	644 (576, 716)	78 (69, 88)	16.9 (14.1, 20.2)	16.6 (13.8, 19.8)	2.01 (1.67, 2.41)	1.12	1.4
Guatemala, all	189	676 (586, 775)	89 (77, 102)	15.8 (12.4, 19.8)	15.4 (12.0, 19.5)	2.02 (1.58, 2.54)	1.12	5.8
Guatemala, BF	141	636 (569, 704)	84 (75, 94)	13.3 (11.1, 15.8)	13.0 (10.8, 15.4)	1.71 (1.44, 2.01)	1.12	4.6
Guatemala, non-BF	48	828 (710, 955)	107 (92, 123)	25.5 (21.2, 29.7)	25.1 (20.6, 29.5)	3.24 (2.68, 3.79)	1.12	0.3
Bangladesh-1, BF	70	564 (501, 628)	75 (68, 84)	10.0 (8.6, 11.5)	9.9 (8.5, 11.3)	1.32 (1.16, 1.49)	1.12	19.1
Aged 12–17 mo								
Peru, all	97	723 (626, 824)	82 (71, 93)	19.8 (15.7, 24.5)	19.0 (15.0, 23.6)	2.15 (1.71, 2.64)	0.95	0.6
Guatemala, all	177	756 (660, 860)	91 (80, 103)	18.2 (14.3, 22.8)	17.4 (13.4, 22.0)	2.09 (1.66, 2.60)	0.95	1.1
Guatemala, BF	117	719 (649, 793)	89 (79, 99)	15.8 (13.2, 18.8)	15.2 (12.6, 18.0)	1.87 (1.58, 2.19)	0.95	0.3
Guatemala, non-BF	60	841 (706, 980)	97 (83, 112)	24.0 (19.0, 29.3)	22.8 (17.3, 28.8)	2.64 (2.04, 3.28)	0.95	0.8
Aged 12–23 mo								
Ecuador, non-BF	248	869 (760, 985)	91 (79, 104)	26.8 (22.7, 31.4)	25.1 (20.7, 29.9)	2.64 (2.16, 3.17)	0.85	0
Uganda, non-BF	47	962 (804, 1128)	95 (81, 109)	24.4 (19.6, 29.7)	20.0 (15.6, 24.9)	1.97 (1.55, 2.43)	0.85	1.1
Aged 24–35 mo								
Ecuador, non-BF	316	887 (772, 1009)	80 (70, 91)	27.5 (23.4, 32.1)	25.6 (21.1, 30.5)	2.32 (1.91, 2.77)	0.79	0.1
Bangladesh-2, all	222	809 (688, 941)	77 (66, 88)	19.2 (15.7, 23.2)	13.5 (11.0, 16.6)	1.28 (1.06, 1.55)	0.79	4.5
Bangladesh-2, BF	140	762 (648, 877)	74 (64, 84)	17.8 (14.7, 21.2)	13.1 (10.6, 15.9)	1.27 (1.05, 1.52)	0.79	4.8
Bangladesh-2, non-BF	82	902 (786, 1026)	83 (73, 94)	22.1 (18.6, 25.9)	14.5 (11.8, 17.6)	1.33 (1.11, 1.60)	0.79	2.9
Uganda, non-BF	121	1219 (1038, 1418)	104 (90, 120)	30.5 (24.9, 36.8)	23.4 (18.6, 28.9)	2.00 (1.59, 2.48)	0.79	0.4
Zambia, non-BF	107	1318 (1079, 1613)	111 (91,136)	40.8 (32.5, 50.5)	29.6 (23.4, 36.9)	2.51 (1.99, 3.10)	0.79	0.2

1 Values are medians (25th percentiles, 75th percentiles). BF, breastfed; EAR, Estimated Average Requirement.

2 See reference 6.

Breastfeeding Bangladeshi-1 infants (aged 6–8 mo) had the lowest median protein intake, with a median intake of 8.9 g available protein/d (1.27 g · kg body weight^−1^ · d^−1^), and the highest prevalence of inadequate available protein intake (24.4%) on a per-kilogram body weight basis (Table 3). A low intake of available protein was also evident in the other cohorts of infants aged 6–8 mo in Peru (15.7%) and Guatemala (12.0% for breastfeeding infants). The prevalence of low available protein intake values decreased by 9–11 mo of age, although less so in Bangladesh-1. The prevalence of low available protein intake values was ≤5% in all children after 12 mo of age.

The percentages of protein intake from 3 sources—breast milk, animal-source foods, and plant-based foods—are depicted in **Supplemental Figure 1**. Among breastfeeding children aged 6–8 mo, the majority of protein was from breast milk, particularly in Bangladesh-1 where the children had the lowest intake of complementary foods, especially animal-source food. Among older infants, children in Peru consumed the greatest amount of protein from plant-based foods (primarily wheat), whereas children in Guatemala had a higher protein intake from animal-source foods (primarily cow milk). Children aged 24–35 mo had greater protein intake from plant- than animal-based foods, except for Ecuadorian children, who consumed the greatest amount of protein from cow milk. The plant-based foods that were the highest sources of protein (expressed as percentage of plant protein) in the diets of children aged 24–35 mo were rice in Bangladesh (66%), groundnuts (21%) and beans (18%) in Uganda, groundnuts (31%) and maize (30%) in Zambia, and rice in Ecuador (18%).

The percentage of children with amino acid intake below the EAR was highest for lysine, although the prevalence of low histidine intake was similarly high for breastfeeding infants aged 6–8 mo (**Supplemental Table 1**). A low intake of sulfur amino acids, threonine, and valine was also evident in children aged 6–8 mo. The prevalence of low amino acid intake was greatly reduced by 9–11 mo of age, and children aged ≥12 mo had mostly adequate amino acid intake amounts.

The dietary intake of infants aged 6–8 mo with and without low available-protein intake was compared to explore whether differences in certain dietary components primarily explained the low intake (**Table 4**). In all 3 cohorts (Peru, Guatemala, and Bangladesh-1), children with a low protein intake had a mean intake of total energy that was 170–212 kcal/d lower, and a mean energy from complementary foods that was 36–227 kcal/d lower, than children with an adequate intake (*P* < 0.0001). Only the Bangladeshi children with a low protein intake also had lower breast milk energy and protein intake than did children with adequate protein intake. Infants with low protein intake had a lower intake of protein from both animal- and plant-source complementary foods. Infants with an adequate protein intake in Peru and Guatemala had a higher protein density (expressed as g · 100 kcal^−1^ · d^−1^) of complementary food intake (*P* < 0.0001), although the median protein density of complementary foods in the low protein groups in all 3 cohorts was near or greater than the proposed recommended protein density of complementary foods of 2 g · 100 kcal^−1^ · d^−1^ (1, 2).

**TABLE 4:** Characteristics of children aged 6–8 mo with an available protein intake less than and greater than or equal to the EAR^1^

	Peru			Guatemala			Bangladesh-1		
	<EAR (*n* = 44)	≥EAR (*n* = 247)	*P*	<EAR (*n* = 32)	≥EAR (*n* = 191)	*P*	<EAR (*n* = 17)	≥EAR (*n* = 36)	*P*
Age, mo	7.3 ± 0.8	7.5 ± 0.8	0.22	6.7 ± 0.9	6.9 ± 0.9	0.33	7.6 ± 0.8	7.7 ± 0.8	0.30
Weight, kg	7.8 ± 1.0	7.6 ± 0.8	0.05	7.4 ± 0.8	6.9 ± 0.8	0.003	6.6 ± 1.0	6.9 ± 0.8	0.63
Length, cm	66.0 ± 2.0	66.0 ± 1.9	0.98	64.5 ± 2.0	64.1 ± 2.5	0.35	65.8 ± 2.8	66.1 ± 2.7	0.83
Length-for-age *z* score	−1.44 ± 0.52	−1.58 ± 0.60	0.15	−1.39 ± 0.82	−1.70 ± 0.96	0.08	−1.59 ± 1.20	−1.38 ± 1.02	0.45
Energy intake									
Total, kcal · kg^−1^ · d^−1^	55 ± 10	80 ± 20	<0.0001	58 ± 11	93 ± 21.9	<0.0001	61 ± 11	87 ± 16	<0.0001
Total, kcal/d	431 ± 80	602 ± 144	<0.0001	426 ± 90	638 ± 151	<0.0001	400 ± 81	596 ± 107	<0.0001
Breast milk, kcal/d	355 ± 93	364 ± 144	0.69	328 ± 137	303 ± 229	0.55	319 ± 123	479 ± 113	<0.0001
Complementary food, kcal/d	77 ± 85	238 ± 213	<0.0001	98 ± 115	335 ± 279	<0.0001	81 ± 81	117 ± 101	0.002
Protein intake									
Total, g · kg^−1^ · d^−1^	0.96 ± 0.12	2.01 ± 1.02	<0.0001	0.91 ± 0.15	2.18 ± 1.14	<0.0001	1.01 ± 0.14	1.52 ± 0.31	<0.0001
Total, g/d	7.6 ± 1.3	15.1 ± 7.5	<0.0001	6.7 ± 1.3	14.9 ± 7.7	<0.0001	6.7 ± 1.3	10.4 ± 2.3	<0.0001
Breast milk, g/d	5.7 ± 1.5	5.9 ± 2.3	0.69	5.1 ± 2.2	4.8 ± 3.6	0.69	5.1 ± 2.0	7.6 ± 1.8	<0.0001
Complementary food, g/d	1.8 ± 1.2	9.3 ± 8.9	<0.0001	1.6 ± 1.7	10.1 ± 10.2	<0.0001	1.6 ± 1.8	2.8 ± 2.9	0.0009
Animal-source food, g/d	0.8 ± 0.8	6.5 ± 7.8	<0.0001	0.7 ± 1.3	7.9 ± 9.9	<0.0001	0.0 ± 0.1	0.5 ± 2.0	0.29
Plant-source food, g/d	1.0 ± 1.1	2.8 ± 2.9	<0.0001	0.9 ± 1.3	2.2 ± 2.8	<0.0001	1.6 ± 1.8	2.3 ± 2.5	0.007
Density of food, g · 100 kcal^−1^ · d^−1^	3.0 ± 1.6	4.0 ± 1.5	<0.0001	1.8 ± 1.3	2.7 ± 1.1	<0.0001	1.8 ± 0.9	2.1 ± 1.1	0.14

1 Values are means ± SDs. The EAR is 1.12 g/kg body weight for infants aged 6–8 mo (6). EAR, Estimated Average Requirement.

The simulated impact of protein digestibility factors that were 5% and 10% lower than the fecal digestibility factors was minor (**Supplemental Table 2**). For example, the estimated prevalence of inadequate protein intake in Peruvian children aged 6–8 mo increased from 15.7% to 15.8% and 16.2% when the digestibility factors were reduced by 5% and 10%, respectively.

## Discussion

The prevalence of dietary protein intake values below the EAR ranged from a high of 24% in Bangladeshi infants aged 6–8 mo to a low of ≤1% in nonbreastfeeding children in Guatemala (aged 9–17 mo), Ecuador (aged 12–35 mo), Uganda (aged 24–35 mo), and Zambia (aged 24–35 mo). The very low prevalence of dietary protein inadequacy in nonbreastfeeding children was apparently the result of the high content of protein in cow milk compared with breast milk. Among breastfeeding children, low protein intake values were primarily because of a low intake of complementary foods during the early stage of transitioning to solid foods (at 6–8 mo of age), and, in the case of Peru and Guatemala, these same cohorts of children had increased their protein intake and had very low evidence of dietary protein inadequacy by 9–11 mo of age. It is not possible to make regional comparisons because of the limited number of studies, the lack of nationally representative data, and differences in availability of data with regard to breastfeeding children.

The finding that low protein intake was because of a low intake of complementary feeding rather than inadequate breastfeeding or low protein densities (or quality) of complementary foods is informative. Other studies have concluded that, unlike specific micronutrients, protein was not a problem nutrient in complementary foods (1–3). This conclusion was mainly based on the protein density of complementary foods when using a proposed recommended protein density of complementary foods of 2 g · 100 kcal^−1^ · d^−1^ for infants aged 6−9 mo (1, 2). With the use of our method of assessing total protein intake adjusted for protein quality, we also conclude that protein inadequacy in the children’s diets was not generally a problem, but there were some children consuming inadequate protein from complementary foods because they were consuming low amounts of complementary foods, particularly in Bangladesh. The authors of that study concluded that the children’s protein intake exceeded the recommended amounts based on the mean of each child’s crude protein intake as a percentage of the Recommended Nutrient Intake, which is set at 2 SD above the EAR (1), being >100% (11). However, this does not provide information on the adequacy of individual children’s diets or the proportion of infants who had an intake below the requirement. When the proportion of infants with an intake below the EAR was estimated in the present analyses, a moderate proportion of the infants had an estimated available protein intake below the EAR (15–24%). It is unclear how the results from this small sample of Bangladeshi infants in 1999 would compare with the situation now, as child malnutrition continues to decline in Bangladesh. In all of the infant cohorts, the improvement in protein intake adequacy during the 9- to 11-mo age period suggests that complementary feeding improved with increasing age for many of the children, although less so in Bangladesh.

The protein quality of complementary foods did not seem to be a major issue with breastfeeding children in the present analyses. The PDCAAS of the diets of the breastfeeding children <24 mo of age was high (>90%). This is due to the high amount of essential amino acids in breast milk and the digestibility of breast milk protein, which compensates for the poorer quality of protein in plant-based complementary foods. The older children who were not breastfeeding and were consuming a high proportion of protein from plant sources had a lower PDCAAS (averaging from 65% to 88%). These Bangladeshi children were consuming a large proportion (45%) of their protein from rice, which is ∼7% protein by dry weight (7% of energy), but it is possible that other populations that were consuming staple foods with very low protein content—such as cassava or sweet potato, which contain <2% protein by weight (3% and 7% of energy, respectively)—could have had a higher inadequacy of protein if their diets were not supplemented with animal-source foods and other staples with higher amounts of protein. In Uganda, sweet potatoes were the major source of energy in the children’s diets (17%), but the protein from nuts and beans, maize, and small amounts of fish were high enough to ensure protein adequacy. The Zambian children had a PDCAAS of 72%, but their total intake of crude protein was high (42 g/d) and legumes and nuts were a major source of total energy (24%) and total crude protein (38%) in their diets.

The finding that lysine was generally the amino acid with the highest prevalence of inadequacy was expected, because lysine is known to be low in staple grains such as wheat, maize, and rice. The intake of histidine was similarly low in the 6- to 8-mo age group, but by 9–11 mo of age, most of the children had an adequate intake of all amino acids except for the Bangladesh-1 children, who still had a low intake of lysine and histidine.

The prevalence of inadequate amino acid intake is generally lower than the prevalence of inadequate protein intake. For example, in the cohort of Peruvian children aged 6–8 mo, 9% had an inadequate lysine intake and 16% had an inadequate protein intake. This is due to the fact that digested amino acid intake is compared directly with amino acid requirements, but the calculation of PDCAAS involves truncation of the amino acid score. In our analyses, we truncated the PDCAAS (which is calculated as the product of the weighted digestibility and amino acid score) rather than the amino acid score, but this made only a slight difference. Our method is mathematically equivalent to the newer suggested method of the digestible indispensable amino acid score, which uses ileal digestibility for individual amino acids (or, if not available, uses the fecal digestibility factors), but this method also includes truncation. If we performed our analyses without any truncation, the estimated available protein intake would be higher than the crude protein intake, which is not plausible.

Our analyses are limited in their representativeness because of a lack of nationally representative population samples and availability of quantitative information on breast milk intake. Some of the cohorts had small sample sizes that may not be optimal for estimating the usual intake distributions of a population. We could not compare our findings directly with other data in the literature because the use of the PDCAAS has not been widely applied. Our analyses did not account for potential higher requirements of protein because of infections, and although infections are frequent in young children in LICs, the degree to which infections increase protein needs is uncertain (7). Our estimates of available protein may also be underestimated because of the potential for decreased nitrogen use when energy intake is low (25). The estimates of protein requirements have changed over time, and there still remains some uncertainty about the digestibility of protein in mixed diets and the additional protein needed to support the growth of malnourished children (7). Nevertheless, we have used the latest recommendations to assess protein adequacy and quality of diets (5, 7), and the simulated impact of lower digestibility of protein did not alter the overall prevalence of inadequate protein intake.

Overall, the protein intake of most children in these cohorts was above estimated protein requirements, except for the younger breastfeeding children. The true test of whether protein intake is adequate for children is to determine whether protein is limiting growth; however, such analyses are difficult to interpret with observational data because of the coexistence of dietary inadequacies of other nutrients that affect growth. Given the high prevalence of stunting in these cohorts and the low prevalence of inadequate protein intake, it seems unlikely that low protein intake was a primary factor explaining linear growth restriction. Moreover, during the age range of the studies, the prevalence of dietary protein inadequacy declined with age, whereas the prevalence of stunting increased. Although many of the present studies were conducted over a decade ago, poor growth and inadequate complementary feeding practices remain prevalent in many LICs and have been shown to be associated with each other (26). The present analyses revealed that many children had a low energy intake from complementary foods, which explained the low protein intake, and this observation reinforces the continued need to promote improved complementary feeding of young children in LICs (27, 28).

## Footnotes

6 Abbreviations used: EAR, Estimated Average Requirement; LAZ, length-for-age *z* score; LIC, low-income country; NCI, National Cancer Institute; PDCAAS, protein digestibility–corrected amino acid score.
